# Heart Failure and a Large Ventricular Thrombus Following COVID-19 Infection

**DOI:** 10.3390/jcdd13030139

**Published:** 2026-03-13

**Authors:** Jouni Taavitsainen, Ville Vepsäläinen, Juha Hartikainen, Jarkko Hytönen

**Affiliations:** Heart Center, Kuopio University Hospital, 70210 Kuopio, Finlandjuha.hartikainen@pshyvinvointialue.fi (J.H.)

**Keywords:** heart failure, COVID-19, ventricular thrombus

## Abstract

Severe cases of heart failure (HF), both new onsets of HF and acute exacerbations of chronic HF, are frequently observed during infections. A potentially lethal complication of HF with very low left ventricular ejection fraction is thrombus formation within the heart chambers. A 67-year-old male was admitted to our hospital with shortness of breath after a COVID-19 infection. He was diagnosed with severe acute heart failure and a massive thrombus in the left ventricle. While the thrombus subsided quickly without any observable embolic events, the patient had a lengthy hospitalization stay complicated by tachyarrhythmias and secondary infections. Eventually, his heart failure improved, and he continued to recover post-hospital discharge. We present a case of severe heart failure and intraventricular thrombosis following COVID-19 infection. The patient required potent anti-inflammatory medication in addition to conventional heart failure medication to recover from his HF.

## 1. Introduction

Heart failure with reduced ejection fraction (HFrEF) is a common indication for hospitalization, with over one million yearly admissions in the US and the EU [1,2]. In this paper, we present a case of acute heart failure following COVID-19 infection with serious complications requiring a lengthy hospital stay.

## 2. Case Presentation

A 67-year-old male was admitted to the emergency department due to worsening dyspnea. He had undergone surgery for bladder cancer one year prior and had been treated with cisplatin and gemcitabine adjuvant therapy before the surgical intervention. He also had a history of iritis and temporal arteritis treated with corticosteroids two years earlier. In addition, he had chronic obstructive pulmonary disease (COPD) and was an active smoker. The patient had no history of heart disease. He had been diagnosed with a COVID-19 infection two months prior to admission to the emergency department. Since the onset of the COVID-19 infection, he had suffered from shortness of breath.

**Figure 1:**
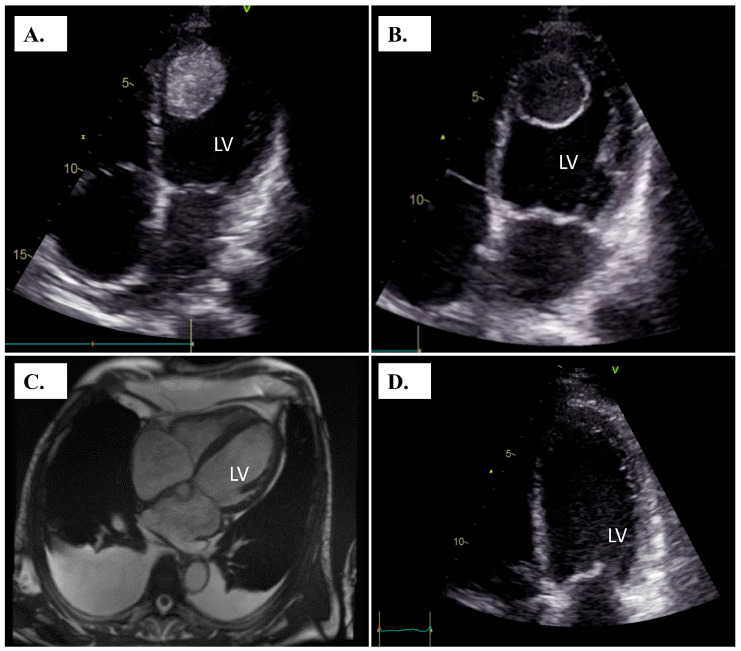
Echocardiogram at the time of hospitalization reveals a large mass in the apex of the left ventricle (**A**). Three days later, the morphology of the mass had changed with a denser core surrounded by a shell with a brighter signal (**B**). One week later, the cardiac MRI showed no evidence of left ventricular thrombus (**C**). No thrombi were seen later during the hospitalization or during the first outpatient visit (**D**). LV = left ventricle.

Prior to hospital admission, the patient was evaluated at the local health center. His chest X-ray showed pleural fluid accumulation, and the pleural fluid had been drained, which temporarily relieved the dyspnea. However, two days later, he developed aggravated dyspnea, nausea, and vomiting, and he arrived at the emergency department of our hospital. He did not present with swelling in the ankles or feet but had an elevated proBNP level at 25,000 ng/L. Bedside transthoracic echocardiography (TTE) revealed reduced left ventricular ejection fraction (LVEF) of 7% and an abnormal mass of 4 × 3.5 cm in the apex of the left ventricle (Figure 1A). The patient had sinus tachycardia of 122 b.p.m. There was no pleural effusion on X-ray at the time of hospitalization. ECG on admission showed tachycardic sinus rhythm, normal conduction times (PQ 148 ms, QRS 88 ms, QTc 415 ms) and T-inversions in V4–V6 but no ST-level abnormalities (Supplementary Figure). Troponin levels (TnT) were slightly elevated at 45 ng/L and remained constant during the hospital stay. Inflammatory markers were low at hospital admission (CRP 7 mg/L, WBC 9.3 × 10^9^/L).

The patient was admitted to the cardiac care unit (CCU), and low molecular weight heparin treatment was started with a reduced dose of enoxaparin 40 mg s.c. twice daily due to moderately-severely decreased glomerular filtration range (creatinine 166 µmol/L, GFR 36 mL/min/1.73 m^2^). The LMWH dose was adjusted according to anti-FXa values with the intention of reaching sufficient levels of anticoagulation. The patient was not hypotensive with a blood pressure of 120-138/88-103 and was subsequently treated with intravenous levosimendan infusion (0.05 µg/kg/min starting dose increased up to 0.2 µg/kg/min, 24 h infusion in total). An oral SGLT-2 inhibitor was initiated shortly after admission in addition to an ACE-inhibitor. Beta-blocker therapy was initiated later during the relatively long hospital stay as his condition improved. TTE control three days after CCU admission showed a change in the consistency of the LV mass. The center of the mass appeared more liquid on echocardiography (Figure 1B). Coronary artery angiogram revealed normal coronaries. The patient developed short episodes of atrial tachycardia (Supplementary Figure) and non-sustained paroxysms of ventricular tachycardia (NSVT), which were treated with intravenous infusions of metoprolol and amiodarone.

The patient’s renal failure subsided, GFR improved to 52 during the first week, and cardiac magnetic resonance imaging (cMRI) was performed on the seventh day after admission. MRI was postponed until the patient’s kidney function had improved due to concerns regarding administration of contrast agents during the early days of hospitalization [3]. Surprisingly, the cMRI showed no signs of the LV thrombus (Figure 1C). The cMRI showed subendocardial enhancement most profoundly in the anterior LV wall but also observed more diffusely within the LV wall. In addition, a T2-weighted signal indicative of edema was observed. The patient had no symptoms or clinical signs of any arterial thromboembolic events.

With poor response to standard heart failure medication and levosimendan, and with indication of a possible inflammatory mechanism responsible for the heart failure based on cMRI, and since the patient had recently been infected with COVID-19, endomyocardial biopsies were collected, and pulse steroid treatment was initiated. After 12 days in the hospital, the patient was given methylprednisolone 1000 mg on three consecutive days. The biopsy samples confirmed lymphocytic myocarditis-the samples were positive for inflammatory cells (CD3, CD45 and CD68), and the histological findings were consistent with myocarditis, although no viral genome PCR testing was available. A week later, prednisolone was initiated with a dose of 60 mg daily. Tests for respiratory viruses were negative apart from the COVID-19 infection.

Two weeks after admission, the patient developed atrial flutter. Cardioversion was performed but it was unsuccessful. Fast atrial flutter of 140 b.p.m. resulted in rapid worsening of the patient’s heart failure. His LVEF had recovered up to 25% but declined to 10% during the tachyarrhythmia. The patient was admitted to the intensive care unit (ICU) due to heart failure and multi-organ failure. Pulse steroid treatment was repeated 30 days after hospitalization. Due to renal failure and acidosis, hemodialysis was initiated in the ICU. With the exception of betablockers, the patient’s heart failure medication was withheld during his stay at the ICU due to hypotension.

The patient was transferred back to the CCU three days after admission to the ICU, during which sinus rhythm had returned, and the patient started to recover from his heart failure while standard heart failure medication was resumed. Hemodialysis was continued for an additional two weeks, after which the patient’s kidney function had improved, and diuresis recovered. During this time, the patient was treated at the cardiac ward. Anticoagulation was initiated during the hospital stay with enoxaparin, followed by tinzaparin, and finally continued with apixaban.

After spending 54 days in the hospital, the patient developed a high fever and became hypotensive. Blood cultures showed Gram-negative growth, which was later confirmed as growth of Klebsiella aerogenes and Serratia marcescens. Piperacillin/tazobactam was started, and he was readmitted to the ICU for two days without needing vasoactive agents. After six days, antibiotic treatment was switched to ciprofloxacin and continued for a further seven days. The patient developed thrombocytopenia, which required thrombocyte transfusions, and the peroral prednisolone dose was increased after hematological consultation. Thrombocyte levels returned to normal with no clear etiological explanation for the thrombocytopenia.

The patient continued to improve clinically, and drug therapy for heart failure was gradually titrated. After 80 days in the hospital, a VVI-ICD pacemaker was implanted. The patient had had paroxysms of atrial fibrillation, atrial flutter, as well as NSVT. In addition, his LVEF was still low at 25% at the time of pacemaker implantation. Taking into account the prolonged hospital stay, the severe heart failure and observed arrhythmias, the decision was made to implant a primary prevention ICD during the hospital stay. The patient was discharged 89 days after hospital admission with an LVEF of 30%. Prednisolone was continued with a daily dose of 20 mg (Table 1). With the patient mainly in sinus rhythm, no plans for ablation of atrial fibrillation or atrial flutter were made during hospitalization.

The first outpatient evaluation was performed four weeks after hospital discharge. The patient was feeling well and was able to climb up 27 stairs without symptoms at home. He reported no shortness of breath, chest pain or palpitations. LVEF was 33%, and there was no sign of thrombi in the cardiac chambers (Figure 1D). Prednisolone dose was further reduced to 15 mg daily with the intention of further reducing the dose during the recovery process. The patient’s ejection fraction had improved significantly, and valsartan/sacubitril treatment was not initiated as he might not have been eligible for drug reimbursement under the Finnish system. Follow-up was scheduled two months after the first outpatient clinic control.

## 3. Discussion

Patients with COVID-19 infection have been shown to develop new onsets of heart failure in up to 23% of hospitalized patients [4]. In addition, pneumonia has been known to influence the coagulation system, exposing patients to thrombotic events [5,6]. Previously, more than sixty cases of COVID-19-related cardiac thrombotic events have been reported with varying risk factors, patient age and severity of outcomes. Left ventricular thrombus has been associated with up to 22% risk of embolization and a 37% risk of major adverse cardiovascular events [7,8]. COVID-19-related myocarditis has been shown to develop with a delay after the actual viral infection, frequently still causing a fulminant disease [9] with patients requiring short-term mechanical support due to severe cardiogenic shock [10].

Anticoagulation has traditionally been accomplished with warfarin, although recent evidence suggests that direct oral anticoagulants are non-inferior to warfarin treatment [11]. The duration of anticoagulation should be determined according to individual patient characteristics, but usually treatment is recommended for at least 3–6 months [7]. Thrombotic events have been documented even after the apparent resolution of the thrombus–something to keep in mind when determining the length of the anticoagulation treatment [12]. For our patient, permanent anticoagulation was warranted regardless of the LV thrombus because of his paroxysmal atrial fibrillation and atrial flutter (CHA2DS2-VASc two points, one for heart failure and one for age 65–74).

The large thrombotic mass changed in morphology and completely disappeared remarkably fast after the initiation of anticoagulant medication. The occurrence of thrombosis is related to Virchow’s triad, as a function of endothelial dysfunction, blood stasis or hypercoagulability. Our patient likely developed thrombus as a result of blood stasis caused by the markedly reduced LV EF and hypercoagulability related to inflammation. The rapid resolution of the LV thrombosis was surprising but similar findings have been reported in case studies [13]. The thrombus has likely either resolved completely with anticoagulation or has caused an asymptomatic embolization.

The severity of myocarditis correlates weakly with elevated troponin values, as was observed with our patient, nor are there necessary abnormal changes in the ECG, although 85% of myocarditis cases present with ECG abnormalities [14]. Endomyocardial biopsies are often needed in cases of severe heart failure and complications such as arrhythmias when an inflammatory mechanism is considered likely, although warrants careful evaluation of the associated risks and benefits [15]. For our patient, an inflammatory cause behind the severe heart failure was deemed likely enough to justify EMB and eventually pulse steroid treatment. In addition, new cases of heart failure typically require standard etiological investigations, including imaging of coronary arteries [2].

Due to the patient’s age and history of malignancy, he was not considered to be a candidate for a heart transplant. Left ventricular assist device (LVAD) was not considered as an option due to the large ventricular thrombus. With persisting, severe heart failure almost two weeks into hospitalization and evidence of inflammatory mechanisms on cMRI, early endomyocardial biopsies were collected, and pulse steroid treatments started. Steroid treatment is indicated for severe cases of lymphocytic myocarditis as described in current ESC guidelines [16].

## 4. Conclusions

Here, we’ve described a severe case of heart failure complicated by a left ventricular thrombus following COVID-19 infection and lymphocytic myocarditis. Our patient likely benefited from an early initiation of pulse steroid treatment, as conventional treatment options were ineffective. In addition to myocarditis, the patient’s heart failure was exacerbated by his tachyarrhythmias and infection during the hospital stay.

## Figures and Tables

**Table 1:** Heart failure medication at hospital discharge.

Active Substance	Concentration	Dosage
bisoprolol	5 mg	bid
ramipril	2.5 mg	bid
apixaban	2.5 mg	bid
dapagliflozin	10 mg	od
prednisolone	20 mg	od
furosemide	20 mg	prn

## Data Availability

The datasets presented in this article are not readily available because they are based on private medical records.

## Supplementary Materials

The following supporting information can be downloaded at: https://www.mdpi.com/article/10.3390/jcdd13030139/s1, Figure S1: ECGs at admission and during tachyarrhythmias.

## Abbreviations

The following abbreviations are used in this manuscript:

HF	Heart failure
HFrEF	Heart failure with reduced ejection fraction
TTE	Transthoracic echocardiography
LVEF	Left ventricular ejection fraction
