# Supplementary figures and images for: Heart Failure and a Large Ventricular Thrombus Following COVID-19 Infection
Source: J Cardiovasc Dev Dis. 2026 Mar 13;13(3):139. doi: 10.3390/jcdd13030139 (PMC13026141; doi:10.3390/jcdd13030139)

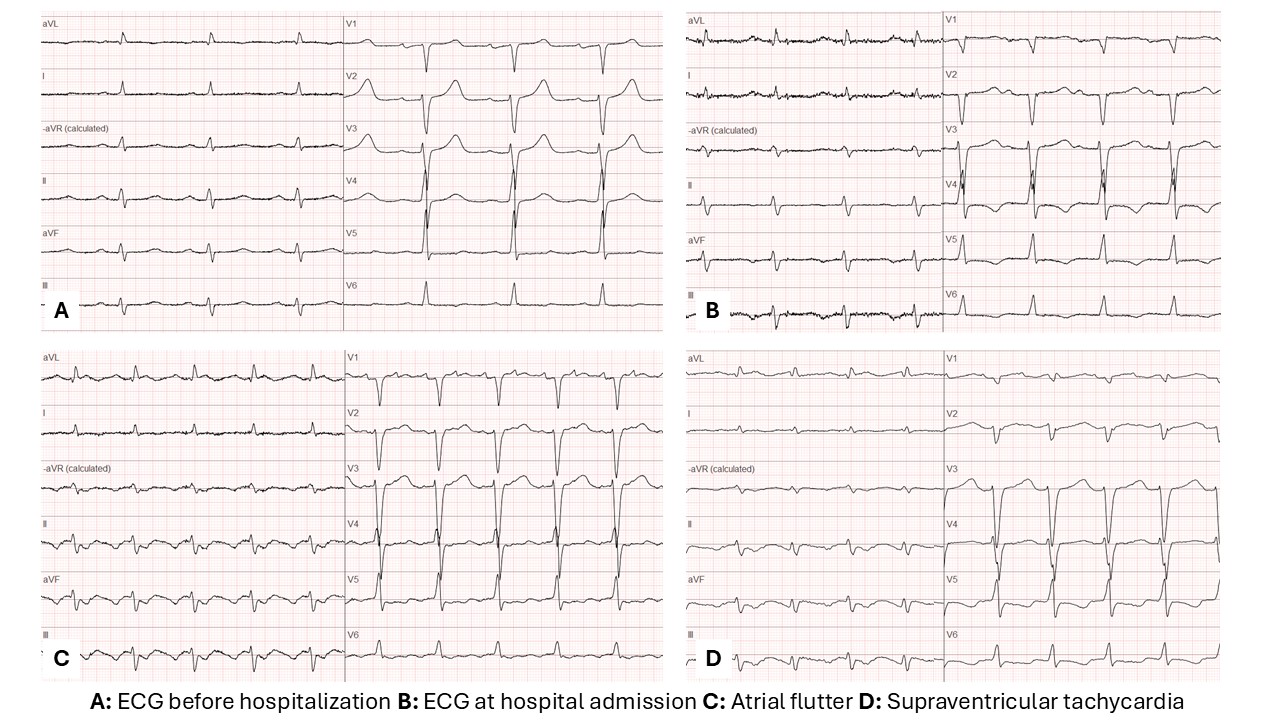

Supplement: Supplementary file 1 [file jcdd-13-00139-s001.zip › jcdd-4157451-Figure S1.JPG]
